# The association between attention-deficit/hyperactivity (ADHD) symptoms and self-employment

**DOI:** 10.1007/s10654-016-0159-1

**Published:** 2016-05-13

**Authors:** Ingrid Verheul, Wim Rietdijk, Joern Block, Ingmar Franken, Henrik Larsson, Roy Thurik

**Affiliations:** 1 Rotterdam School of Management, Erasmus University Rotterdam, P.O. Box 1738, 3000 DR Rotterdam, The Netherlands; 2 Erasmus School of Economics, Erasmus University Rotterdam, Rotterdam, The Netherlands; 3 Institute for Behavior and Biology (EURIBEB), Erasmus University Rotterdam, Rotterdam, The Netherlands; 4 Professur für Unternehmensführung, Universität Trier, Trier, Germany; 5 Erasmus Research Institute of Management (ERIM), Erasmus University Rotterdam, Rotterdam, The Netherlands; 6 Institute of Psychology, Erasmus University Rotterdam, Rotterdam, The Netherlands; 7 Montpellier Business School, Montpellier, France; 8 Department of Medical Epidemiology and Biostatistics, Karolinska Institutet, Stockholm, Sweden; 9 Karolinska Institutet Center of Neurodevelopmental Disorders (KIND), Stockholm, Sweden

**Keywords:** Attention-deficit, Hyperactivity, Symptoms, ADHD, Self-employment

## Abstract

Attention-deficit/hyperactivity (ADHD) symptoms have been associated with the decision to become self-employed. Although these symptoms are generally regarded as disadvantageous, there may also be a bright side. To our knowledge, however, there has been no systematic, epidemiological evidence to support this claim. This paper examines the association between ADHD symptoms and self-employment in a population-based sample from the STAGE cohort of the Swedish Twin Registry (N = 7208). For replication, we used a sample of Dutch students who participated in the Global University Entrepreneurial Spirit Students’ Survey (N = 13,112). In the Swedish sample, we found a positive association with self-employment for both general ADHD symptoms [odds ratio (OR) 1.13; 95 % confidence intervals (CI) 1.04–1.23] and hyperactivity symptoms [OR 1.19; 95 % CI 1.08–1.32], whereas no association was found for attention-deficit symptoms [OR 0.99; 95 % CI 0.89–1.10]. The positive association between hyperactivity and self-employment was replicated in the Dutch student sample [OR 1.09; 95 % CI 1.03–1.15]. Our results show that certain aspects of ADHD, in particular hyperactivity, can have a bright side, as they are positively associated with self-employment.

## Introduction

Prominent entrepreneurs have publicly credited their attention-deficit hyperactivity (ADHD) symptoms as a driver of their decision to become self-employed [1–3]. Examples include Ingvar Kamprad (founder of IKEA) and Richard Branson (founder of the Virgin Group) [2, 3]. The Economist recently published a short article addressing the suitability of self-employment for people who experience ADHD symptoms [4].

ADHD is a neurodevelopmental disorder characterised by attention-deficit and hyperactivity [5–7]. The onset of ADHD is typically during childhood (before the age of 12), and it is therefore often regarded as a childhood disorder. Studies following children with a diagnosis of ADHD have shown that ADHD symptoms are persistent over time, at least into early adulthood [6, 8–11], with 65 % of individuals still having a full ADHD diagnosis or partial remission at the age of 25 [12]. Recent research has devoted more attention to increasing our understanding of the persistence of ADHD symptoms in (young) adults [13]. Furthermore, the most recent DSM criteria were adjusted to enable a diagnosis across the lifespan and not only during childhood [14].

Although prior research has mainly focused on the negative consequences of ADHD for individual performance in formal education and wage employment [15–19], recent studies have highlighted positive aspects of ADHD symptoms, such as its association with resilience and well-being [20], close friendship [21], and third-party inferences about an individual’s generative qualities (being creative, visionary, good at generating ideas) [22]. In the present study, we focus on a potential positive aspect of ADHD: self-employment as a career choice. Self-employment is not just any career choice but is a manifestation of entrepreneurship that is essential for employment creation, innovation and the economic growth of modern societies [23, 24]. It can be used as an economic instrument in the business cycle [25] and to lift people out of unemployment [26].

In the popular press, it has been suggested that individuals with ADHD symptoms are able to break through organisational inertia due to their ability to envision and create new ‘realities’ and (successfully) start their own ventures [2–4]. Allegedly, when individuals who experience ADHD symptoms succeed in developing mechanisms to cope with their ‘weaknesses’, they would be able to exploit their extraordinary talents and perform just as well as or even better than their peers in business [2, 27, 28]. Academic research has found only circumstantial evidence linking ADHD symptoms to self-employment when examining the association with entrepreneurial intentions [29], creativity [30–32], risk-taking [33], and proactivity [5]. To our knowledge, there is no systematic, epidemiological evidence supporting a link between ADHD symptoms and self-employment.

The present study was designed to examine the association between self-reported ADHD symptoms and self-employment in a population-based sample of 7208 individuals from the STAGE cohort of the Swedish Twin Registry of the Karolinska Institute. The findings were replicated in a sample of 13,119 students participating in the Global University Entrepreneurial Spirit Students’ Survey (GUESSS) 2011 in the Netherlands. Given the discussions in the (popular) literature and the circumstantial scholarly evidence, we expected a positive association between ADHD symptoms and the decision to become self-employed [1–4, 29]. We should emphasise that the present study does not focus on ADHD as a full-blown psychiatric disorder. For the purpose of the present research, self-reported psychiatric symptoms are defined along a continuum [34] and not as a psychiatric classification.

## Method

### Swedish twin registry: STAGE cohort as the discovery sample

We used the STAGE (Swedish Twin Studies on Adults: Genes and Environment) cohort from the Karolinska Institute in Stockholm, Sweden, as the discovery sample. The STAGE cohort is part of the population-representative Swedish Twin Registry (STR), which was established in the 1950s to study the effects of smoking and drinking on cancer and cardiovascular diseases [35]. At present, the STR contains rich data about biological and clinical markers together with the socio-economic background of twins living in Sweden [35]. For a full account of the design and execution of the STR project and details of the ADHD data in the STAGE cohort, we refer to Lichtenstein et al. [35] and Larsson et al. [10], respectively.

All twins born between 1959 and 1985 (a total of 42,582 twins) received an invitation letter with information about the project in May and June 2005. The response rate was 59.6 % (N = 25,364, 56 % female, age[mean] = 41.56, age[sd] = 7.6). The entire questionnaire contained approximately 1300 questions, of which the respondents answered 800–900 questions on average [35]. Dropouts could be explained by a general unwillingness to respond to a lengthy survey [10]. Non-respondents were significantly more often male, had at least one parent born outside Sweden, and were more likely to have been diagnosed with a psychiatric disorder and convicted of any type of crime [10, 36, 37], which may indicate that the most severe cases did not respond to the questionnaire. This limits generalisations to the more severe cases of the ADHD spectrum. Informed consent was obtained from all individual participants included in the study. This project was reviewed and approved by the ethics committee of the Karolinska Institute.

### Self-employment measure

Participants answered two questions about their involvement in self-employment: whether they were self-employed full-time (yes/no) and whether they were self-employed part-time (yes/no). We constructed one measure of self-employment (yes/no), which was coded 1 if the participant was self-employed part-time or full-time and 0 if (s)he was not self-employed. In the STAGE cohort, 14,039 out of 25,364 participants (≈55 %) provided their self-employed status (yes/no). A total of 2096 participants (≈14 %) were self-employed, of whom 1270 (≈9 %) were self-employed full-time and 826 (5 %) were self-employed part-time. Three participants answered ‘yes’ to both the part-time and full-time self-employment question and were excluded from the analysis. Furthermore, we randomly dropped one twin from each pair because their similarity would violate the assumption of independent observations, resulting in a final sample of 7208 participants (58 % female, age[mean] = 43.9, age[sd] = 6.7). Of these respondents, a total of 897 (≈12 %) were self-employed, of whom 515 (≈7 %) were self-employed full-time and 382 (≈5 %) were self-employed part-time, which was similar to the total sample distribution.

### Attention-deficit/hyperactivity (ADHD) symptoms measure

ADHD symptoms were assessed using the Adult ADHD Self-Report Scale (ASRS-v1.1), which consists of 18 items reflecting the DSM-IV-TR criteria [10]. Attention-deficit symptoms and hyperactive symptoms were measured by 9 items each.^1^ Answers for each item were given on a 3-point Likert scale (0 = ‘no’; 1 = ‘yes, to some extent’; and 2 = ‘yes’). The 18 ASRS-v1.1 items were slightly modified to allow for the assessment of ADHD symptoms in adults [35]. As can be expected from a non-clinical population sample, many participants reported having no or few ADHD symptoms. The average percentage of respondents reporting inattentive symptoms and hyperactive symptoms (with the answer ‘yes’) was 3.6 and 4.6 %, respectively. Approximately one-third of the respondents reported no symptoms [10]. This result closely resembles the prevalence rate of adult ADHD reported in Kessler et al. [38].

The symptom scores were added to create a scale of ADHD symptoms and two sub-scales of attention-deficit and hyperactivity [10]. Values for the reliabilities of these three scales in our sample were α = 0.84, α = 0.78, and α = 0.78, respectively. These reliabilities were similar to those reported in Larsson et al. [10]. Because the three scales were highly skewed (skewness: ADHD, 1.66; attention-deficit, 1.77; hyperactivity, 1.72), the scales were log-transformed (Log10[x + 1], where x is the initial value) to normalise their distributions (skewness after log transformation: ADHD, -0.16; attention-deficit, 0.27; hyperactivity, 0.26). As indicated in Larsson et al. [10, p. 199], the sum score of the 18 ADHD items used in this study yielded results similar to those reported in other studies using self- or parent-reported measures of ADHD.

### Control variables

In the STAGE cohort, we included the following demographic variables: age, gender, and whether the participant attended university (0 = no, 1 = yes) as an initial test to control for additional effects.

### GUESSS study: replication

We attempted to replicate the analysis linking ADHD symptoms to self-employment in a student sample from the Global University Entrepreneurial Spirit Students’ Survey (GUESSS)^2^ 2011 in the Netherlands. GUESSS is an international research consortium that studies the career objectives of students in higher education. In the Netherlands, students at 14 universities and 24 polytechnics received a link to the online survey through email. After 1 month, a reminder was sent. Two randomly drawn participants received an iPad 2.0 to reward their participation. To prevent self-selection of students with entrepreneurial intentions, the topic of the survey was announced as future career paths. The GUESSS Netherlands study was in line with the Erasmus Research Institute of Management review board’s standards and did not include clinical or patient data [29].

The response rate for the GUESSS study was 7.6 % for educational institutions that systematically recruited participants [29], leading to an overall database of 13,119 students (56 % female, age [mean] = 22.96, age [sd] = 0.49), of whom 374 were student entrepreneurs (i.e., reported having their own business during their studies). Comparing the GUESSS Netherlands sample with the global GUESSS sample reported in Sieger et al. [39], we found that our (Dutch) sample was representative in terms of socio-demographic characteristics and the prevalence of student entrepreneurs. Specifically, in our sample, 56 % of the respondents were female compared to a global average of 55.2 %, and the average age of 23 was within the mid-range age of the international average, which is between 20 and 27 years [39]. Furthermore, 2.9 % of the students in our study had their own company, compared to a global average of 2.5 % student entrepreneurs [39].

In the GUESSS study, self-employment was measured as a dichotomous variable indicating whether students were self-employed in their own founded firm during their studies. ADHD symptoms were measured using the six-item Adult ADHD Self-Report Scale v1.1 (ASRS v1.1) screener, developed by Kessler et al. [38, 40], which consists of the six most predictive ADHD symptoms of the 18 DSM-IV-TR criteria used in the STAGE study. This enabled us to replicate the analysis in the STAGE cohort. Each item was answered on a 5-point Likert scale (1 = never to 5 = always). Although the measures were different, the screener and the full 18-item measure were found to be highly correlated [38, 40]. Average scores were computed for ADHD symptoms (based on six items, α = 0.59), attention-deficit (based on four items, α = 0.62), and hyperactivity (based on two items, α = 0.43). Although these scales had low to moderate reliability, they were still close to the lower bound of reported reliabilities for the ASRS screener questions in Kessler et al. [40] (see Verheul et al.) [29]. The three scales were only moderately skewed: sample skewness for the ADHD symptom scale, the attention-deficit scale, and the hyperactivity scale amounted to 0.21, 0.35, and 0.06, respectively, with a standard error of 0.02. These skewness measures indicated that log-transformation of the variables was not necessary. Given that the GUESSS study consisted of only students from universities and polytechnics, we only included age and gender as control variables in the model.

### Statistical analysis

To examine the association between ADHD symptoms and self-employment, we estimated binary logistic regressions. First, in the STAGE cohort, we examined the association between the sum score on the DSM-IV diagnostic criteria items for all ADHD symptoms (18 items), attention-deficit and hyperactivity symptoms (each 9 items), and self-employment (part-time/full-time). Second, we replicated this analysis using data from the GUESSS study. Specifically, we estimated the associations between the sum score on the ASRS-6 v1.1 screener items for all ADHD symptoms (6 items), attention-deficit symptoms (4 items), hyperactivity symptoms (2 items), and self-employment. We report the effect sizes in terms of odds ratios (OR) and the associated 95 % confidence intervals (95 % CI). In the tables, we denote statistical significance (*p* values at 5, 1, and 0.1 % levels, respectively) using asterisks (*, **, and ***, respectively).

## Results

### STAGE cohort

In Table 1, we present the results of the binary logistic regressions explaining self-employment (part-time/full-time). We found a positive association with self-employment for both general ADHD symptoms (OR 1.13; 95 % CI 1.04–1.23) and hyperactivity symptoms (OR 1.19; 95 % CI 1.08–1.32) but not for attention-deficit symptoms (OR 0.99; 95 % CI 0.89–1.10).

**Table 1:** STAGE cohort; Binary logistic regressions with self-employment (part-/full-time) as dependent variable and general ADHD symptoms, and the two sub-scales attention-deficit symptoms and hyperactivity symptoms as independent variables

	(1)	(2)
General ADHD symptoms	1.13**	
	(1.04–1.23)	
Attention-deficit symptoms		0.99
		(0.89–1.10)
Hyperactivity symptoms		1.19***
		(1.08–1.32)
N	7208	7208
Log-likelihood	−2599	−2597
df	4	5
Chi-square	209.2	222.7
Pseudo R-square	0.04	0.04

The coefficients are odds ratios (ORs) and 95 % confidence intervals (CI) are in parentheses; *** *p* < 0.001, ** *p* < 0.01, * *p* < 0.05

Both models control for age, gender, and university education (0 = no and 1 = yes)

### GUESSS study: replication

We attempted to replicate the association between ADHD symptoms and self-employment using the GUESSS 2011 data. The results of the binary logistic regressions are presented in Table 2. We found no evidence of an association between general ADHD symptoms and self-employment (OR 1.00; 95 % CI 0.97–1.02). However, when we separated attention-deficit and hyperactivity, we found a negative association between self-employment and attention-deficit symptoms (OR 0.95; 95 % CI 0.92–0.99) and a positive association between self-employment and hyperactivity symptoms (OR 1.09; 95 % CI 1.03–1.15).

**Table 2:** GUESSS study; Binary logistic regressions with self-employment (part-/full-time) as dependent variable and general ADHD symptoms, and the two sub-scales attention-deficit symptoms and hyperactivity symptoms as independent variables

	(1)	(2)
General ADHD symptoms	0.99	
	(0.93–1.07)	
Attention-deficit symptoms		0.89*
		(0.79–1.00)
Hyperactivity symptoms		1.13*
		(1.00–1.28)
N	13,119	13,119
Log-likelihood	−3189	−3183
df	3	4
Chi-square	208.5	214.8
Pseudo R-square	0.07	0.07

The coefficients are odds ratios (ORs) and 95 % confidence intervals (CI) are in parentheses; *** *p* < 0.001, ** *p* < 0.01, * *p* < 0.05

Both models control for age, and gender

To summarise, we found a positive association between (general) ADHD symptoms and self-employment in the STAGE cohort that could not be replicated (i.e., insignificant) in the GUESSS study. Furthermore, the association between attention-deficit symptoms and self-employment was significantly negative in the GUESSS study and insignificant in the STAGE cohort. Finally, the association between hyperactivity symptoms and self-employment was significantly positive in both the STAGE cohort and the GUESSS study.

### Sensitivity and additional analyses

We performed a series of sensitivity analyses using both the STAGE and GUESSS data. First, we reran the logistic regressions using the entire STAGE database, including pairs of twins instead of randomly dropping one twin, and clustered the standard error at the family level to account for the joint characteristics of twins. In the larger sample of approximately 11,000 respondents, the association between ADHD symptoms and self-employment remained intact (OR 1.23; 95 % CI 1.13–1.34). Furthermore, the results of the sub-scales remained stable: attention-deficit was not significantly associated with self-employment (OR 1.02; 95 % CI 0.94–1.11), and hyperactivity was positively associated with self-employment (OR 1.19; 95 % CI 1.10–1.29).^3^

Second, to compare the results of the GUESSS study with those of the STAGE cohort, we examined the associations between ADHD symptoms and self-employment in the STAGE cohort using the 6 items from the ASRS-6 v1.1 screener instead of the 18 item ASRS-v1.1 score. In line with the GUESSS findings, we found no evidence of a significant association between general ADHD symptoms and self-employment (OR 1.04; 95 % CI 1.00–1.09). However, we did not find support for a significant (negative) association between self-employment and attention-deficit symptoms (OR 0.97; 95 % CI 0.9–1.03). We did find a positive association between hyperactivity symptoms and self-employment (OR 1.04; 95 % CI 1.00–1.09), which is in agreement with the analyses.^4^

Third, we examined the association between ADHD symptoms and self-employment using a stricter definition of ADHD that was closer to an actual psychiatric diagnosis [10]. In the STAGE data, we used the “strict criteria” score: in the responses to the DSM-IV 18 items, 1 (“yes”) was recoded to yes and 2 or 3 (“yes, to some extent” or “no”) were recoded to no. The outcomes supported those of the initial analysis. The association between ADHD symptoms and self-employment became stronger (OR: 1.35; 95 % CI 1.00–1.81), which was also true for hyperactivity (OR: 1.54; 95 % CI 1.03–2.29). In the GUESSS data, for each of the ASRS-6 v1.1 items, we examined whether the score was beyond the threshold level (‘yes = 1’ and ‘no’ = ‘0’) and constructed new scale variables for ADHD symptoms, attention-deficit and hyperactivity based on the sum of the dummy (0,1) scores on six, four and two items, respectively. The findings remained stable for ADHD symptoms (not significant, OR: 1.00; 95 % CI 0.93–1.07), attention-deficit (significant, OR: 0.90; 95 % CI 0.82–0.98), and hyperactivity (significant, OR: 1.27; 95 % CI 1.11–1.47).

Fourth, we re-estimated the logistic regressions including additional control variables. Specifically, because unemployment is an important push factor for self-employment [26] and because individuals who experience ADHD symptoms have a higher chance of unemployment [38, 41], we added a control variable in the analyses with the STAGE data that captured whether the individual had mainly been unemployed or hardly worked in the 3 years preceding the survey. The association between ADHD and self-employment remained positive and significant (OR: 1.15; 95 % CI 1.06–1.25). This was also true for hyperactivity (OR: 1.19; 95 % CI 1.07–1.32). Furthermore, we controlled for personality characteristics of people with ADHD symptoms. We included measures for extraversion and neuroticism to account for the fact that ADHD symptoms have been linked to (higher) neuroticism [42, 43] and extraversion [44]. These Big Five Factor traits have also been linked with self-employment [45, 46]. When controlling for these two personality factors, both general ADHD symptoms (OR: 1.17; 95 % CI 1.06–1.29) and hyperactivity (OR: 1.14; 95 % CI 1.02–1.28) remained significantly positive. In the analyses with the GUESSS data, we were able to control for factors that are generally associated with self-employment status, including self-employed parents, study field and level. In particular, parental role models have been linked with the decision to become self-employed [47, 48]. Accounting for the aforementioned factors in the analyses, the results were stable, and only attention-deficit and hyperactivity were significantly associated with self-employment (attention-deficit, OR: 0.96; 95 % CI 0.92–0.99; hyperactivity, OR: 1.07; 95 % CI 1.01–1.13).

Overall, the association between ADHD symptoms and self-employment remained intact but became insignificant when more controls were added. In the STAGE data, this was the case when considering whether someone was mainly wage employed in the past 3 years. This is not surprising given the fact that approximately 60 % of the respondents were currently wage employed and had mainly been working in wage jobs in the past 3 years. In the GUESSS data, the association between ADHD symptoms and self-employment appeared sensitive to the inclusion of career motives (e.g., challenge, realising a dream, income, financial security, innovation, opportunity exploitation, high position, flexibility, own boss, social mission), which may be related to the importance of the person-occupation fit for individuals with ADHD symptoms [29].

Finally, using the STAGE data, we tested for linkages between ADHD symptoms and other occupations. The findings showed no association with wage employment (OR: 0.96; 95 % CI 0.90–1.03), suggesting that a regular wage job may not be as suitable as self-employment for people with high levels of ADHD symptoms. Together with our finding that ADHD symptoms are positively associated with unemployment (OR: 1.24; 95 % CI 1.12–1.37), we are unable to reject a potential push effect by which people with ADHD symptoms become self-employed because of a lack of (suitable) alternative occupations.

## Discussion

The present study moved beyond the clinical view of ADHD as a pathological disorder and used the symptoms of ADHD across the entire measurement spectrum to examine a potentially positive aspect: the association of ADHD with the involvement in self-employment. Hence, the aim of this study was not to diagnose individuals with ADHD and then examine the viability of self-employment as a career option. Instead, we investigated whether individuals who exhibit higher levels of ADHD symptoms–but who do not necessarily screen positive for ADHD in a clinical sense–are positively associated with self-employment. In line with previous research, special attention was paid to whether this association involved general ADHD symptoms or the separate attention-deficit and hyperactivity symptom dimensions [10, 34, 49–51].

The present research is part of a series of investigations on the relation between ADHD and entrepreneurship. In Verheul et al. [29], the intention to become self-employed was found to be associated with ADHD symptoms in a survey of Dutch students. In Thurik et al. [52], the entrepreneurial orientation, measured using innovativeness, risk aversion and proactivity [53], was found to be associated with ADHD symptoms in a survey of French business owners. The present study investigates involvement in self-employment. All three studies report a positive association, although the hyperactivity part seems to dominate. Future studies should also take into account entrepreneurial performance, such as growth, profits, longevity, and satisfaction.

Two independent samples were used for the present analysis: Swedish adults (STAGE cohort) and Dutch students (GUESSS study). In the Swedish sample, we found a positive association of both general ADHD symptoms and hyperactivity symptoms with self-employment, whereas this association could not be found for attention-deficit symptoms. The positive association between hyperactivity symptoms and self-employment was replicated in the Dutch sample. The effects remained intact in a series of sensitivity analyses.

ADHD is typically characterised by high energy levels that are expressed as severe and persistent attention-deficit and hyperactivity and that are driven by behavioural ‘disinhibition’ or a lack of restraint [22, 41, 54]. Less attention is paid to ADHD symptoms for adult decision-making [55], but it is generally recognised that high levels of attention-deficit and hyperactivity have negative consequences in the workplace. Individuals who demonstrate such behaviours tend to show low job performance and a high chance of becoming unemployed [56–58]. However, the present results show that ADHD symptoms, particularly hyperactivity, are associated with aspects that may be beneficial for the individual and society at large.

The present study has implications for further research. Our results may be an initial step towards establishing a link between ADHD symptoms and career choices, such as self-employment, or other manifestations of entrepreneurship. The outcomes of this study may help to ‘destigmatise’ ADHD as a disorder, particularly given the positive associations with self-employment in view of its contribution to socio-economic life. Given the high occurrence of moderate psychiatric symptoms, it is plausible (from a Darwinian perspective) that psychiatric symptoms not only confer risks but can also be beneficial for the individual. For the field of psychopathology, it is important to study the potential benefits of demonstrating a high level of ADHD symptoms [21, 32, 59, 60] across the lifespan [11, 14, 27, 61]. Such a focus on the value (rather than the cost) of ADHD is at the heart of a recent stream of literature in the field of psychiatry (i.e., Darwinian Psychiatry), which argues that the persistence of such mental ‘disorders’ serves a purpose [27, 61–63]. According to this research stream, psychiatric symptoms or genetic variations that are mostly or currently disruptive for an individual’s work and private life can–under some circumstances or in mild forms–be beneficial for the ‘adaptation’ or survival of the individual. Hence, (young) adults who experience mild to severe ADHD symptoms may benefit rather than suffer from them provided they find ways to cope with the negative consequences in general and in relation to an entrepreneurial context [22]. Benefits may be particularly salient when individuals with ADHD symptoms find a suitable work environment, such as self-employment, where the ‘disorder’ is not harmful but instead can be valuable and help them to function well in society [21, 27, 61].

Although the present study only investigates one single outcome, self-employment as a possibly positive aspect of ADHD symptoms, it highlights some promising avenues for future research. First, the current data do not enable us to examine the association between ADHD symptoms and the performance of self-employed individuals. The question that arises is whether self-employed individuals who score higher on ADHD symptoms also have better-performing ventures [53]. Second, the decision to become self-employed may not be the only association with ADHD symptoms; underlying entrepreneurial behaviours such as risk-taking and proactivity may share this association [52]. ADHD symptoms may also have ‘positive’ associations with other socio-economic behaviours for occupational choice, such as in management and consultancy positions [24]. Third, to generalise the results of this study, it is worthwhile to examine the association between ADHD symptoms and self-employment in other, preferably non-European, population-based cohorts. It is also important to distinguish between individuals with a different occupational status in the control group, including wage employment and unemployment. A first attempt is reported in our section on sensitivity analyses. For future research, it is important to distinguish between these (alternative) occupations to effectively examine the association with different control groups.

To conclude, our results indicate that the positive association between ADHD symptoms and the decision to become self-employed primarily hinges on the hyperactivity symptoms of ADHD, whereas the overall association between ADHD symptoms and self-employment is only significant in one of our two samples. For future research, it is important to understand how ADHD symptoms are associated with more specific self-employment and entrepreneurship behaviours, such as the level of risks taken or performance in self-employment or as a business owner. This research may enhance our understanding of the positive effects of ADHD symptoms or even ‘destigmatise’ ADHD as a disorder that always deserves treatment.

## Footnotes

1 Throughout the paper we refer to hyperactivity symptoms, although the ASRS v1.1 measure is slightly broader given that it includes three items related to impulsivity.

2 For more information about GUESSS, we refer to http://www.guesssurvey.org.

3 For the analyses including the ADHD symptom scale (plus controls), the log pseudo likelihood was −2705, the pseudo R-square was 0.06, and the Wald Chi-square was 319.4. Standard errors were adjusted for 8434 clusters in pair ID (identifying twins). For the analyses separating attention-deficit and hyperactivity, the log pseudo likelihood amounted to −4118, the pseudo R-square was 0.04, and the Wald Chi-square was 354.8. Standard errors were adjusted for 8771 clusters in pair ID.

4 In line with Kessler et al. [38, 40], the reliability coefficients (Cronbach’s alpha) for general ADHD symptoms, attention-deficit symptoms and hyperactivity symptoms were 0.59, 0.58, and 0.50, respectively, indicating that the scales were moderately reliable.
